# *ADAT3*-related neurodevelopmental disorder in 24 new patients with a high frequency of the p.Val144Met and a new founder variant

**DOI:** 10.1038/s41598-025-06857-2

**Published:** 2025-06-27

**Authors:** Karima Rafat, Asmaa F. Abdel-Aleem, Hasnaa M. Elbendary, Mahmoud Y. Issa, Mona L. Essawi, Sherif F. Abdel-Ghafar, Ghada M. H. Abdel-Salam, Mohamed S. Abdel-Hamid, Maha S. Zaki

**Affiliations:** 1 Department of Clinical Genetics, Human Genetics and Genome Research Institute, National Research Centre, Cairo, Egypt; 2 Department of Medical Molecular Genetics, Human Genetics and Genome Research Institute, National Research Centre, Cairo, Egypt; 3 Clinical Genetics Department, Human Genetics and Genome Research Institute, National Research Centre (NRC), Cairo, 12311 Egypt

**Keywords:** *ADAT3*, Neurodevelopmental disorder, Dysmorphic facies, Novel variants, Egyptian patients, Founder effect, Genetics, Molecular biology, Diseases, Health care, Neurology

## Abstract

Biallelic variants in the adenosine deaminase tRNA specific 3 *(ADAT3)* gene are associated with a distinct neurodevelopmental disorder characterized by dysmorphic facies, poor growth, cognitive impairment, and variable brain anomalies. We describe 24 patients from 16 unrelated Egyptian families with *ADAT3*-related neurodevelopmental disorder. All patients presented with developmental delay, growth retardation, cognitive impairment, and the characteristic facial features of the disorder, which appears to be more recognizable in older patients. Seizures were noted in 20% of patients and showed favorable responses to treatment. Brain imaging showed corpus callosum abnormalities in most patients (91.6%), followed by delayed myelination and cortical atrophy. Exome sequencing identified three *ADAT3* variants, including the Saudi founder variant c.430G > A (p.Val144Met), which was detected in 17 patients (70%). In addition, two novel variants were identified, c.319G > A (p.Glu107Lys) and c.1013_1018dup (p.Arg338_Ile339dup). The c.319G > A (p.Glu107Lys) was recurrent in 6 patients (25%) who shared a similar haplotype, suggesting a likely founder effect in our population. On the other hand, the c.1013_1018dup (p.Arg338_Ile339dup) was identified in a single patient. Our study reports a large cohort of patients with *ADAT3*-related neurodevelopmental disorder from Egypt and reinforces the clinical and brain imaging characteristics of the disorder. The high prevalence of the c.430G > A (p.Val144Met) in our population strongly suggests the existence of a founder effect of this variant in the Middle East and Arab region. In addition, we report a new founder variant expanding the mutational spectrum of this rare disorder.

**Supplementary Information:**

The online version contains supplementary material available at 10.1038/s41598-025-06857-2.

## Introduction

 Adenosine deaminases acting on tRNA (ADATs) are enzymes that modify transfer RNA (tRNA), a key molecule in translating genetic codes into proteins. These enzymes change adenosine to inosine in tRNA, allowing it to pair with multiple codons during protein synthesis, a process vital for cellular function^1^. Humans have three ADAT enzymes (ADAT1, ADAT2, and ADAT3), and each has a distinct role with structural analyses clarifying their specific contributions to tRNA editing^2^. ADAT1 edits tRNA-Ala at position 37, while ADAT2 and ADAT3 form a complex that targets position 34, the wobble position, in several tRNAs, a mechanism critical for translational fidelity under cellular stress^3^. Structural studies of the ADAT2/ADAT3 complex show how ADAT3 stabilizes the enzyme, highlighting its importance in mammals. Disruptions in this process can impair protein synthesis, leading to neurological dysfunction^2^.

The *ADAT3* is located on chr19p13.3 and consists of two exons. The first exon is non-coding while exon 2 codes for 367 amino acids including the highly conserved functional deaminase domain CMP/dCMP-type^4^. Biallelic variants in *ADAT3* are associated with a neurodevelopmental disorder with poor growth, dysmorphic facies, and brain abnormalities (OMIM# 615286). Patients present with a range of clinical features including global developmental delay, microcephaly, hypotonia, growth retardation, and intellectual disability. Strabismus, prominent forehead, up-slanted palpebral fissures, and depressed nasal bridge are characteristic facial features of the disorder. Neurological manifestations such as epilepsy and brain abnormalities are frequently reported alongside behavioral problems. Some cases might show endocrine dysfunction (growth hormone deficiency or adrenal insufficiency) further highlighting the systemic impact of ADAT3 deficiency^4–6^.

To date, a total of 60 patients from 29 unrelated families with *ADAT3* variants have been described in the literature. The c.430G > A (p.Val144Met) is the most commonly reported variant, found in 26/29 families (frequency ~ 90%). The majority of these families were from Saudi Arabia and therefore this variant was denoted as a “Saudi founder variant”^4–13^.

Herein, we present the clinical, brain imaging, and molecular data of 24 new patients with *ADAT3* variants. To our knowledge, this is the first study from Egypt and North Africa presenting a large cohort of patients with *ADAT3*-related neurodevelopmental disorder.

## Patients and methods

### Patients

The study included 24 patients from 16 unrelated families recruited from the Neurogenetics/Neuropediatrics Clinic and the Outpatient Clinic of the Clinical Genetics Department at the National Research Centre (NRC), Cairo, Egypt. The study was approved by the Medical Research Ethics Committee of NRC in accordance with the “World Medical Association Declaration of Helsinki” in 1995 (as revised in Seoul 2008), and written informed consents were obtained from the parents for the study participation and photographs for online open-access publication. All patients were subjected to thorough medical history taking, complete general examination, full neurological assessment, and basic anthropometric measurements, including head circumference, height, and weight. Dysmorphic features were identified and documented by photography. Investigations such as karyotyping, extended metabolic screening, ophthalmological evaluation, auditory brain stem evoked potential, electroencephalogram (EEG), electromyography (EMG), nerve conduction velocity (NCV), and brain MRI were performed. Thyroid function tests and growth hormone, both basal and after stimulation, were also done when indicated. Assessment of intellectual disabilities was done using Stanford-Binet Intelligence Scales version 5^14^.

## Methods

### Extraction of genomic DNA

DNA was extracted from the peripheral blood lymphocytes of the patients and their parents using PAXgene Blood DNA Kit (QIAGEN, Germany) according to the manufacturer’s instructions.

## Exome sequencing

Exome sequencing (ES) was performed for one patient from each family. Exome capture was applied using xGen Exome Research Panel v2 (Integrated DNA Technologies, Coralville, Iowa, USA) and sequencing was performed using NovaSeq 600 (Illumina, San Diego, CA, USA). Approximately 98.9% of the targeted bases were covered to a depth of ≥ 20x. The obtained sequences were aligned to UCSC human genome GRCh37/hg19 and variants were verified using the GATK pipeline. Annotation of variants was performed using the BaseSpace Variant Interpreter Server. Variants were prioritized based on the mode of inheritance, impact on the gene product, minor allele frequency and multiple silico predicted pathogenicity. We focused on rare biallelic variants (new or ≤ 0.001 in public genetic databases) related to the patients’ phenotype.

## Segregation analysis

Segregation analysis of the three identified *ADAT3* variants in the parents and other affected siblings was performed using Sanger sequencing. Primers were designed by Primer3 SOFTWARE. PCR cycling conditions were: initial denaturation at 96 °C for 5 min; 30 cycles of denaturation at 96 °C for 30 s; annealing at 62 °C for 30 s; extension at 72 °C for 30 s, and a final extension at 72 °C for 5 min. PCR products were purified using Exo-SAP PCR Clean-up kit (Thermo Fisher Scientific, Germany) and sequenced in both directions using the BigDye Terminator v3.1 Cycle Sequencing Kit (Applied Biosystems, Foster City, CA, USA) and analyzed on the ABI Prism 3500 Genetic Analyzer (Applied Biosystems) according to manufacturer’s instructions.

### In silico analysis of the novel missense variant

The pathogenicity of the detected novel missense variant was evaluated by various web-based in silico tools including SIFT^15^, DANN^16^, PrimateAI^17^, Polyphen-2^18^, MutationTaster^19^, and REVEL^20^. MetaDome webserver was further checked for the mutation tolerance of the variant^21^. The HOPE project was applied to investigate the variant effect on protein structure **(**HOPE,https://www3.cmbi.umcn.nl/hope/). I-Mutant2.0, MUpro, and DynaMut2 were applied to evaluate the variant impact on the protein stability^22–24^. MusiteDeep was used to predict the possible post-transcriptional modification (PTM) associated with the altered amino acid sequence^25^. RNAfold web server (http://rna.tbi.univie.ac.at//cgi-bin/RNAWebSuite/RNAfold.cgi) was utilized to investigate the change of minimum free energy (MFE) of the mRNA secondary structures of mutant sequence compared to the wild-type.

## Results

The detailed clinical and molecular data of the 24 patients are summarized in Table 1.

**Table 1:** The clinical, brain imaging, and molecular findings of the studied patients with homozygous *ADAT3* variants.

Family number	Family 1	Family 2	Family 3	Family 4	Family 5		Family 6	Family 7	Family 8			Family 9	
Patient number	P1	P2	P3	P4	P5	P6	P7	P8	P9	P10	P11	P12	P13
**Variant**	c.319G> A	c.430G> A p.Val144Met	c.430G> A p.Val144Met	c.430G> A p.Val144Met	c.430G> A p.Val144Met	c.430G> A p.Val144Met	c.430G> A p.Val144Met	c.430G> A p.Val144Met	c.430G> A pzVal144Met	c.430G> A p.Val144Met	c.430G> A p.Val144Met	c.319G> A	c.319G> A
	p.Glu107Lys											p.Glu107Lys	p.Glu107Lys
**Zygosity**	Homozygous	Homozygous	Homozygous	Homozygous	Homozygous	Homozygous	Homozygous	Homozygous	Homozygous	Homozygous	Homozygous	Homozygous	Homozygous
**Age at last examination**	7y 4m	10m	8y	3y 2m	8y	3y	5y	17y	10y	4y	40y	7y	2y
**Sex**	Female	Male	Female	Female	Female	Female	Female	Male	Male	Female	Female	Female	Female
**Consanguinity**	+	+	+	+	+	+	+	+	+	+	+	+	+
**Family history**	−	−	−	−	+	+	−	+	+	+	+	+	+
					(Sister of P6)	(Sister of P5)			(Brother of P10)	(Sister of P9)	(Aunt of P9 and P10)	(Sister of P13)	(Sister of P12)
**Developmental delay**	+	+	+	+	+	+	+	+	+	+	+	+	+
Cognitive impairment /(IQ)*	Severe (35)	Moderate	Severe (30)	Severe (38)	Severe (33)	Severe (35)	Severe (39)	Severe (29)	Severe (34)	Severe (32)	Severe (35)	Severe (28)	Severe (25)
**Behavioral problems**	Panic attacks	−	Autistic features	−	Panic attacks, Masturbation like movement	Autistic features, Masturbation like movement	−	Autistic	Hyperactive,	ADHD,	Hyperactive	Autistic	Autistic
									aggressive, temper tantrum, Masturbation like movement	spit in her hands			
**Speech ability**	Poor (Few words)	−	Absent	Poor (Few words)	Poor (Few words)	Poor (Few words)	Poor (Few words)	Absent	Poor (Few words)	Poor (Few words)	Poor (Few words)	Absent	Absent
**Epilepsy (type)**	−	+ (Myoclonic)	−	−	−	−	−	+ Tonic	−	−	−	−	−
**Recurrent Otitis media**	−	−	−	−	−	−	−	−	−	+	−	+	−
**Anthropometric measurements**													
**HC (cm/SD)**	50.5 (-2.4)	41.5 (-3.5)	47 (-3.7)	46.5 (-2.2)	48 (-2.8)	43 (-3.5)	46 (-3.1)	54 (-0.7)	49 (-2.5)	46.5 (-2.3)	54 (-0.3)	47 (-3.6)	41.5 (-4.2)
**Wt (Kg/ SD)**	14.5 (-3.7)	7.5 (-2.4)	16.5 (-2.3)	10 (-2.7)	20 (-0.9)	11 (-2.1)	8 (-3.1)	35 (-3.3)	24.5 (-0.85)	13.2 (-1.25)	70 (+0.7)	17.5 (-1.9)	8 (-3.7)
**Ht (cm/ SD)**	102 (-3.3)	65 (-3.3)	117 (-1.9)	77 (-4.1)	113 (-2.0)	86.5 (-2.1)	103 (-1.2)	131(-5.4)	133 (-0.6)	91 (-1.25)	162 (-0.19)	100 (-4.1)	73 (-3.6)
**Facial features**													
**Dysmorphic facies**	+	+	+	+	+	+	+	+	+	+	+	+	+
**High forehead**	+	+	+	+	+	+	+	+	+	+	+	+	+
**Prominet forehead**	+	+	+	+	−	+	−	+	+	+	+	+	+
**Strabismus**	+	+	+	+	+	+	+	+	+	+	+	+	+
**Upslanting palpebral fissures**	+	+	+	+	+	+	+	+	+	+	+	−	−
**Epicanthic folds**	+	+	+	+	+	+	+	+	+	+	+	+	+
**Telecanthus**	+	+	+	+	+	+	+	+	+	+	+	+	+
**Depressed nasal bridge**	+	+	+	+	+	+	+	+	+	+	+	+	+
**Prominent nose**	+	+	+	+	+	+	+	+	+	+	+	−	−
**Short nose**	+	+	−	+	−	−	+	+	−	−	−	+	+
**Wide mouth**	−	−	+	+	+	−	+	+	+	+	+	−	−
**Micrognathia**	+	+	−	−	−	+	−	−	−	−	−	+	+
**Full lips**	+	−	+	−	+	+	−	+	+	+	+	+	−
**Teeth abnormalities**	−	−	−	−	−	−	−	−	−	−	−	−	−
**Low-set ears**	+	+	−	−	−	−	−	−	−	−	−	+	+
**Large ears**	−	+	+	+	+	+	+	+	+	+	+	−	−
**Neurological assement**													
**Axial hypotonia**	+	+	+	+	−	−	−	+	−	−	−	+	+
**Limb hypotonia**	+	−	+	+	−	−	−	+	+	+	+	−	−
**Limb hypertonia/Spasticity**	−	+	−	−	+	+	+	−	−	−	−	+ (Mild)	+ (Mild)
**Reflexes**	Present	Brisk	Hyporeflexia	Present	Brisk	Brisk	Brisk	Present	Present	Present	Present	Present	Present
**Brain MRI findings**													
**Cortical atrophy**	+ (Mild)	−	+ (Mild)	−	−	−	−	−	−	−	−	+ (Mild)	+ (Mild)
**Corpus callosum hypogenesis/dysgenesis**	-/-	+/-	-/-	+/-	+/-	+/-	+/-	+/-	+/-	+/-	+/-	+/-	+/-
**Myelination defect**	−	−	+	−	−	−	−	+	+	+	+	+ (Mild)	+ (Mild)
**Reduced white matter volume**	−	−	+	−	−	−	−	+	+	+	+	+ (Mild)	+ (Mild)
**Dilated lateral ventricles**	+	−	+	−	−	−	−	+	+	+	+	−	−
**Cerebellum/ brain stem changes**	−	−	−	−	−	−	−	−	−	−	−	−	−
**Endocrinal abnormalities**													
**Growth hormone deficiency**	+	+	+	+	−	−	−	−	−	−	−	+	−
**Other findings**	−	−	−	−	−	−	Pectus carinatum	−	−	−	−	−	−

Family number	Family 10		Family 11	Family 12		Family 13		Family 14	Family 15		Family 16
Patient number	P14	P15	P16	P17	P18	P19	P20	P21	P22	P23	P24
**Variant**	c.430G > A p.Val144Met	c.430G > A p.Val144Met	c.430G > A p.Val144Met	c.430G > A p.Val144Met	c.430G > A p.Val144Met	c.430G > A p.Val144Met	c.430G > A p.Val144Met	c.1013_1018dup p.Arg338_Ile339dup	c.319G > A p.Glu107Lys	c.319G > A p.Glu107Lys	c.319G > A p.Glu107Lys
**Zygosity**	Homozygous	Homozygous	Homozygous	Homozygous	Homozygous	Homozygous	Homozygous	Homozygous	Homozygous	Homozygous	Homozygous
**Age at last examination**	8y	2y 8m	8y 8m	6y 8m	6y 8m	9y	2y	2y	7y 11m	6y 6m	1y 11m
**Sex**	Male	Female	Male	Female	Female	Female	Female	Male	Male	Female	Male
**Consanguinity**	+	+	+	+	+	+	+	+	+	+	+
**Family history**	+ (Brother of P15)	+ (Sister of P14)	−	+ (Twin of P18)	+ (Twin of P17)	+ (Sister of P20)	+ (Sister of P19)	−	+ (Brother of P23)	+ (Sister of P22)	−
**Developmental delay**	+	+	+	+	+	+	+	+	+	+	+
Cognitive impairment (IQ)*	Severe (38)	Severe (35)	Mild (55)	Severe (35)	Severe (30)	Moderate (45)	Moderate (35)	Severe (30)	Severe (37)	Severe (30)	Moderate
**Behavioral problems**	−	−	−	−	Autistic	Masturbation like movement	−	Irritability, sleep disturbance	−	−	−
**Speech ability**	Poor (Few words)	Poor (Few words)	Poor (Few words)	Poor (Few words)	Poor (Few words)	Poor (Few words)	Poor (Few words)	Poor (Few words)	Poor (Few words)	Absent	Poor (Few words)
**Epilepsy (type)**	−	−	−	−	−	−	−	+ (Tonic with fever)	+ (GTC, 2 months, controlled on levetiracetam)	+ (GTC, 6 months, controlled on levetiracetam)	−
**Recurrent Otitis media**	−	−	+	−	−	−	+	+	−	−	−
**Anthropometric measurements**											
**HC (cm/SD)**	46.5 (-2.5)	43 (-3.7)	48 (-3.6)	48 (-2.7)	48 (-2.7)	51.5 (-0.2)	47 (-0.3)	44 (-3.3)	44 (-6)	44 (-5.9)	43.5 (-3.5)
**Wt (Kg/ SD)**	18 (-1.1)	13 (-0.6)	24 (-1.4)	11 (-2.7)	12 (-2)	20 (-2.3)	11 (-0.9)	7 (-4.6)	14 (-2.8)	9.5 (-3.2)	9 (-2.9)
**Ht (cm/ SD)**	110 (-1.06)	85 (-2.4)	125 (-2.6)	113 (-1.2)	116 (-1)	115 (-2.9)	78 (-2.2)	79 (-2.3)	102 (-3.3)	92 (-4.2)	78 (-2.3)
**Facial features**											
**Dysmorphic facies**	+	+	+	+	+	+	+	+	+	+	+
**High forehead**	+	+	+	+	+	+	+	+	+	+	+
**Prominent forehead**	+	+	+	+	+	+	+	+	+	+	+
**Strabismus**	+	+	+	+	+	+	+	+	+	+	+
**Upslanting palpebral fissures**	+	+	+	−	−	+	+	+	−	+	−
**Epicanthic folds**	+	+	+	+	+	+	+	+	−	−	−
**Telecanthus**	+	+	+	+	+	+	+	+	+	+	+
**Depressed nasal bridge**	+	+	−	+	+	+	+	+	−	−	+
**Prominent nose**	−	−	−	−	−	+	+	+	+	+	+
**Short nose**	+	+	+	+	+	+	+	+	−	−	+
**Wide mouth**	+	+	+	−	−	+	+	+	−	−	−
**Micrognathia**	−	−	−	+	+	−	−	−	+	+	−
**Full lips**	+	+	+	+	+	−	−	−	- (Thin)	- (Thin)	−
**Teeth abnormalities**	−	−	+	−	−	+	−	+ (Delayed eruption)	+ (Macrodontia)	−	−
**Low-set ears**	+	+	−	−	−	+	+	+	−	−	+
**Large ears**	+	+	−	+	+	+	+	+	+	+	+
**Neurological assement**											
**Axial hypotonia**	+	+	−	−	−	−	+	+	−	−	−
**Limb hypotonia**	+	+	−	−	−	−	+	+	−	−	−
**Limb hypertonia/Spasticity**	−	−	+	+	+	Mild hypertonia, flexed knees	−	−	+/+	+/+	−
**Reflexes**	Present	Present	Present	Brisk	Brisk	Brisk	Present	Present	Hyperreflexia	Hyperreflexia	Present
**Brain MRI findings**											
**Cortical atrophy**	−	−	−	+ (Mild)	+ (Mild)	−	+ (Mild)	+ (Mild)	−	−	−
**Corpus callosum hypogenesis/dysgenesis**	-/+	+/-	+/-	+/-	+/-	+/-	+/-	+/-	+/-	+/-	+/- (Complete agenesis)
**Myelination defect**	+	+	−	+	+	−	+	+	+	+	+
**Reduced white matter volume**	+	+	−	+	+	−	+	+	−	−	−
**Dilated lateral ventricles**	+	+		+	+	−	+	+	−	−	−
**Cerebellum/ brain stem changes**	−	−	−	−	−	−	−	−	−	−	Mild CB, BS hypoplasia
**Endocrinal abnormalities**											
**Growth hormone deficiency**	−	−	−	−	−	−	−	+	−	−	−
**Other findings**	−	−	−	−	−	−	−	−	Low birth weight, hydrocele	Low birth weight	−

ADHD: Attention deficit hyperactivity disorder, BS: Brain stem, CB: Cerebellum, GTC: Generalized tonic–clonic seizures, HC: Head circumference, Ht: Height, m: Month, NA: Not available, P: Patient, Wt: Weight, y: Year.

*IQ: Intellectual quotient was done by Stanford Binet (5th edition).

## Clinical data

The patients were 16 females and 8 males and their ages ranged from 10 months to 40 years. Consanguinity was evident in all patients (100%). The presence of similarly affected family members was noted in 16/24 patients (66%). All patients had obvious developmental delay. Severe cognitive impairment was recorded in 19/24 patients (79%). Behavioral problems including autistic features, hyperactivity/attention deficit hyperactivity disorders (ADHD), masturbating-like movement, panic attacks, irritability, and sleep disturbance were documented in 6 patients (25%), 3 patients (12.5%), 4 patients (16.7%), 2 patients (8.3%), 1 patient (4.1%), and 1 patient (4.1%), respectively. Poor and absent speech were noted in 18 patients (75%) and 5 patients (20%), correspondingly. Epilepsy was recorded in 5 patients (20%) and was controlled on treatment with multiple antiepileptic drugs.

**Fig. 1:**
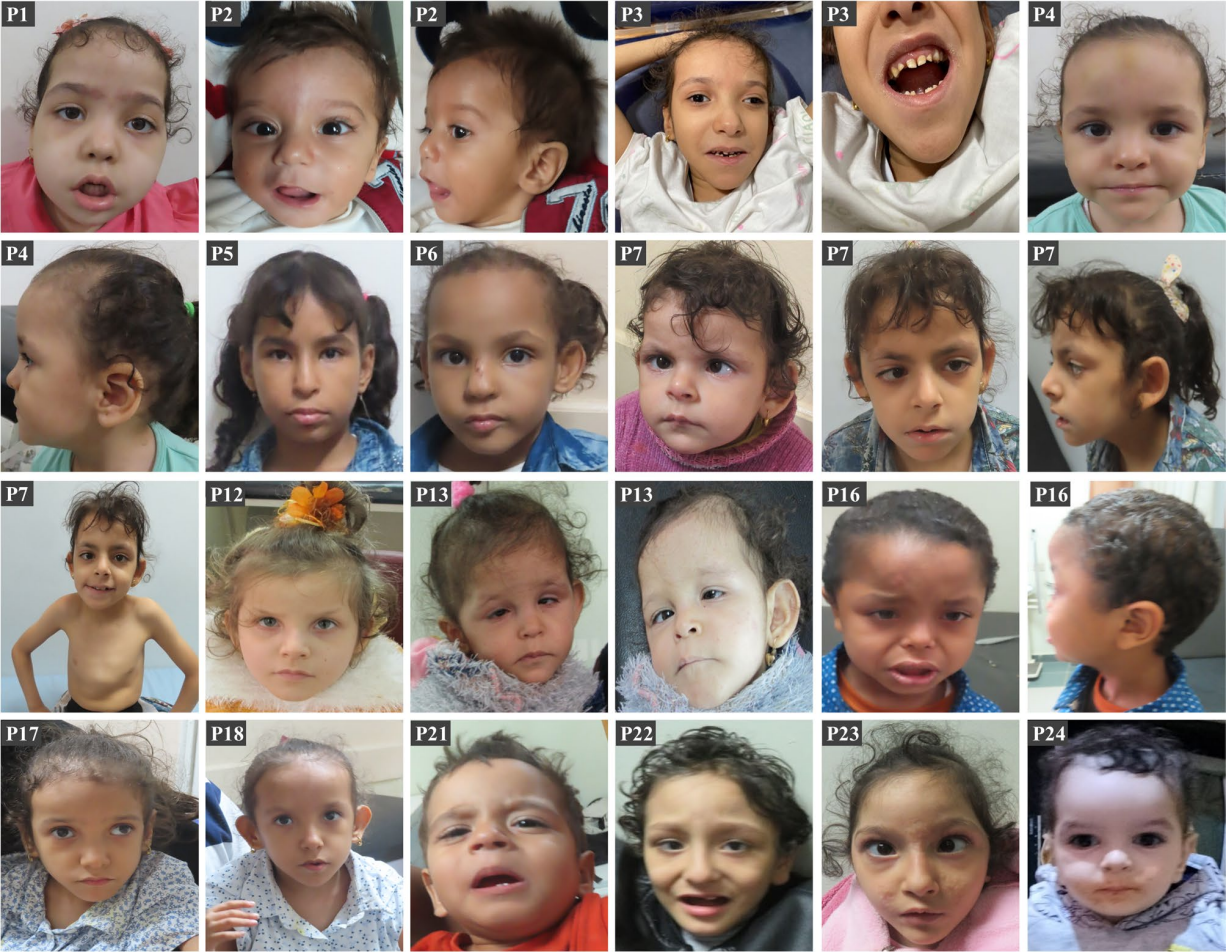
Facial features of our patients. Note the high and prominent forehead, strabismus, epicanthic fold, upward slanting palpebral fissure, prominent nose, micrognathia, large and low-set ears, in addition to abnormal teeth (P3) and pectus carinatum (P7). Also, facial features became more pronounced with age as seen in P7.

Anthropometric measurements showed microcephaly in 12 patients (50%), underweight in 15 patients (62.5%), and short stature in 16 patients (66%). Dysmorphic features were noted in all patients (100%). The most common facial features in the present patients were: high forehead (100%), strabismus (100%), telecanthus (100%), prominent forehead (91%), epicanthic fold (87%), depressed nasal bridge (87%), large ears (83%), upslanting palpebral fissure (75%), prominent nose (70%), short nose (66%), low-set ears (41%), and micrognathia (37%) (Fig. 1). Neurological examinations showed axial hypotonia in 11 patients (45%), limb hypotonia in 11 patients (45%), and limb hypertonia in 12 patients (50%). None of our patients had neuropathy or contractures. Endocrinal dysfunction in the form of growth hormone deficiency was noted in 6 patients (25%). Rare findings observed in one patient each were macrodontia, delayed teeth eruption, and pectus carinatum.

**Fig. 2:**
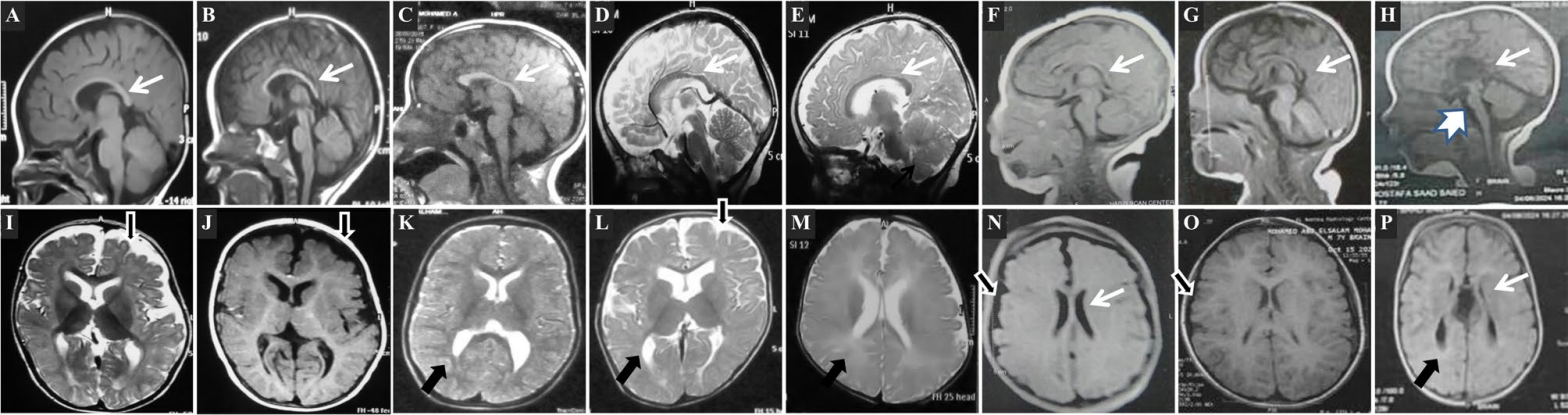
Patients’ MRI: Sagittal view cuts (A-H) showing corpus callosum abnormalities *(thin white arrow)*: thin corpus callosum (A, B, D, E), dysgenesis of corpus callosum (C, F, G), absent corpus callosum (H) and mild hypoplastic vermis and brainstem (H) *(thick white arrow*,* P24*). Axial view cuts (I-P) showing mild cortical atrophic changes (I, J, L, N, O) *(black arrow with a white edge)*, deep white matter changes (K, L, M, P) (*black arrow*), colpocephaly and agenesis of corpus callosum (P) *(white arrow)*, mild dilated lateral ventricles (I, K, L, M).

Neuroimaging findings comprised corpus callosum abnormalities (agenesis, hypogenesis, or dysgenesis) in 22 patients (91%), followed by abnormal white matter, either hypomyelination defect (16 patients; 66.6%) or reduced volume (13 patients; 54%), and mild cortical atrophy (8 patients; 33%). Notably, cerebellar involvement was absent in all but one patient who exhibited mild cerebellar and brainstem hypoplasia in addition to the agenesis of corpus callosum (Pt 24) **(**Fig. 2**)**.

### Molecular data

**Fig. 3:**
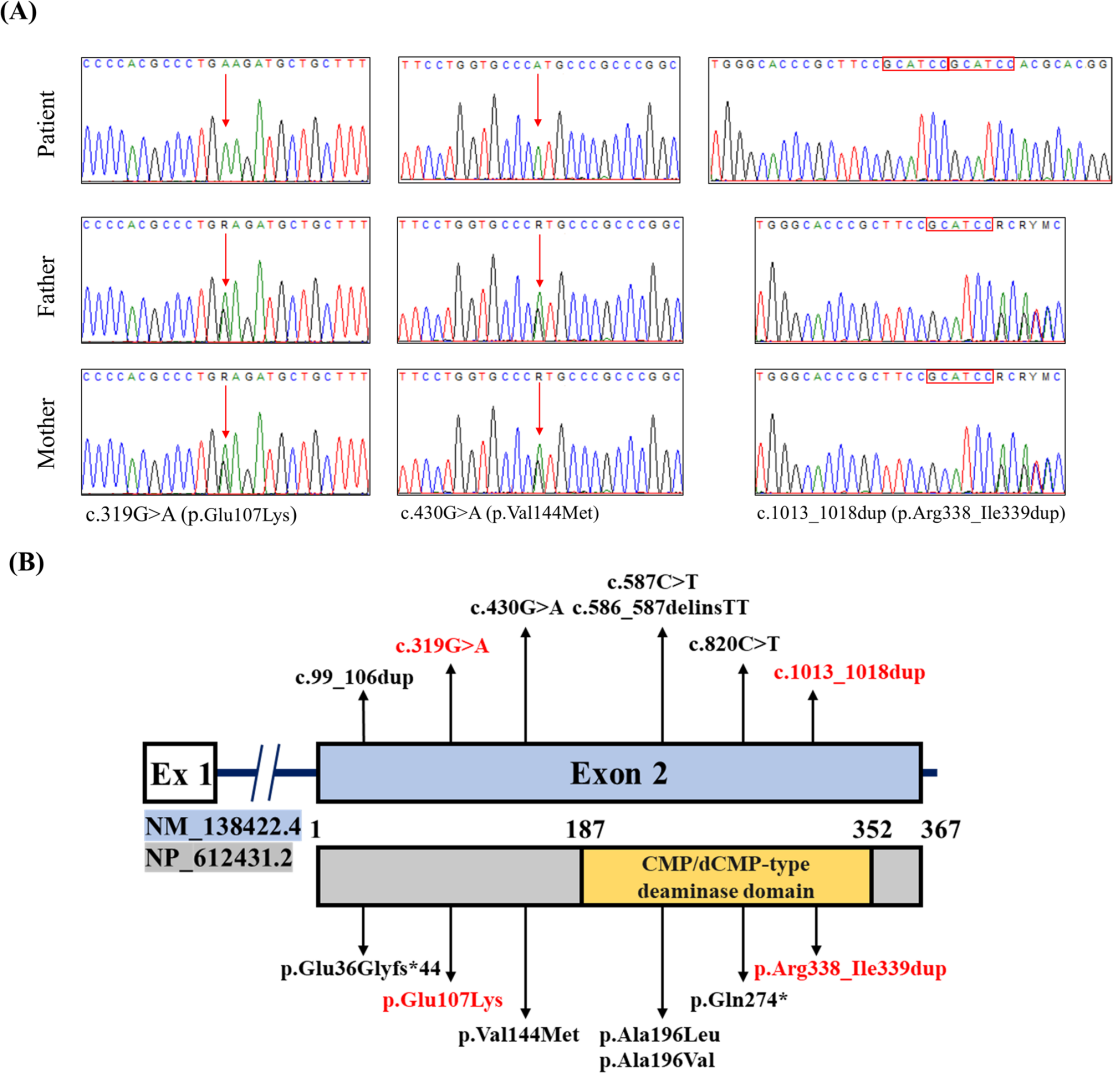
(**A**) Portion of the sequencing electropherograms showing the three *ADAT3* variants identified in our study. The arrow indicates the site of the variant. (**B**) Schematic diagram showing the *ADAT3* coding exon, protein domain, and all reported variants so far, including our two novel variants in red.

Exome sequencing identified three homozygous variants in the *ADAT3* (NM_138422.4) in all patients. The previously reported pathogenic missense variant c.430G > A (p.Val144Met) was detected in 17 individuals from 11 unrelated families (frequency 70%). A new missense variant c.319G > A (p.Glu107Lys) was recurrent in 6 patients from four unrelated families (frequency 25%). In addition, one patient (Patient 21) was found to harbor an in-frame duplication variant, c.1013_1018dup (p.Arg338_Ile339dup). The three variants segregated with the phenotype in all families (Fig. 3A). The c.319G > A (p.Glu107Lys) and c.1013_1018dup (p.Arg338_Ile339dup) are not found in public genetic databases (gnomAD v.4, 1000 genomes databases, GME Variome, or ClinVar) or our inhouse database. According to the American College of Medical Genetics and Genomics (ACMG) classification, the c.319G > A (p.Glu107Lys) should be classified as a “likely pathogenic” variant, and the c.1013_1018dup (p.Arg338_Ile339dup) as a “variant of uncertain significance”. The two variants have been submitted to ClinVar under accession number SCV005373787.

### In Silico prediction of the missense variant

**Fig. 4:**
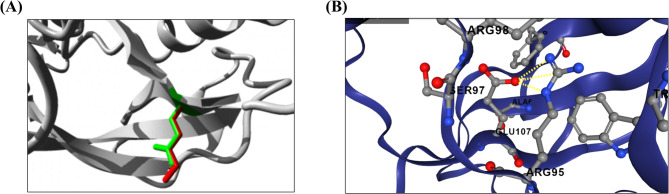
(**A**) Structural variation of the wild and mutant residues in ADAT3 according to the HOPE project. Zooming on the novel detected variant (p.Glu107Lys), the protein is colored gray, and the side chains of the wild type (Glutamic acid) and the mutant (Lysine) residues are colored green and red, respectively. (**B**) Close-up of the salt bridge (represented by yellow dashed lines) between the wild glutamic acid at position 107 and arginine residue at position 95 according to the DynaMut2.

 By using in silico prediction tools, the c.319G > A (p.Glu107Lys) is predicted as deleterious by SIFT, DANN, and PrimateAI while as benign by PolyPhen-2, MutationTaster, and REVEL. MetaDome web server showed that the variant is located in a region slightly intolerant to missense variation. According to HOPE, the protein structure might be affected and disruption of the salt bridge between the wild glutamic acid and arginine residue at position 95 is predicted due to the different size and charge of the mutant lysine. Additionally, MUpro, I-Mutant2.0, and DynaMut2 showed decreased stability of the mutant protein **(**Fig. 4). The MusiteDeep tool predicted methylation of mutant lysine as PTM (threshold: 0.532). mRNA secondary structure of the mutant sequence showed a change in the minimum free energy compared to the wild sequence (MFE= −785, −788)^26^ (**Fig. ****S1**). In silico results are all summarized in (Table 2).

## Discussion

*ADAT3*-related neurodevelopmental disorder is very rare with only 60 patients from 29 unrelated families reported in the literature^4,5,7,8,10–13,27^. Our study reports an additional 24 patients and significantly substantiates the clinical and neuroimaging characteristics. All our patients shared the core clinical features of the disorder, including dysmorphic facies, motor delay, delayed or absent speech, and moderate to severe cognitive impairment. Strabismus, prominent forehead, telecanthus, epicanthic fold, depressed nasal bridge, and upslanting palpebral fissure were the most common facial features identified. These facies are more prominent and appear recognizable in older patients. Microcephaly, growth retardation, and hypotonia were common findings in our patients, similar to previous reports^4,5,7,8,10–13,27^. Although epilepsy is not frequently reported in patients with *ADAT3* variants, it was found in 5 patients of our cohort (20%). Interestingly, the 5 patients responded well to antiepileptic drugs. Unlike previous reports (Table 2), behavioral problems (autism, hyperactivity, ADHD, and panic attacks) were common in our patients and found in 13/24 patients (54%).

**Table 2:** In silico analysis of the novel *ADAT3 *variants.

	Variant in *ADAT3* (NM_138422.4) (nucleotide change, amino acid change, exon)	
	c.319G > A *p*.Glu107Lys Exon 2	c.1013_1018dup *p*.Arg338_Ile339dup Exon 2
**SIFT**	Deleterious	−
**DANN**	Deleterious (1)	−
**PrimateAI**	Deleterious (0.9)	−
**REVEL**	0.12	−
**PolyPhen-2**	Benign	−
**MutationTaster**	Benign	Benign
**MetaDome tolerance**	Slightly intolerant	−
**ClinVar**	No entry	No entry
**GenomAD v.4**	Absent	Absent
**HOPE**	Possible negative effect on protein function	−
**MUpro**	Decrease stability (ΔΔG= −0.97)	−
**DynaMut2**	Decrease stability (ΔΔG= −0.88)	−
**I-Mutant2.0**	Decreased stability	−
**MusiteDeep**	Lysine methylation, threshold=(0.532)	−
**MFE score of secondary mRNA structure**	Wild=−788, Mutant= −785	−
**ACMG Classification**	Likely Pathogenic (PP1-S, PP2, PM2, BP4)	VOUS (PM2, PM4, BP4)

**Table 3:** Clinical and molecular chartacteristics of reported cases with biallelic *ADAT3* variants.

	Alazami et al. (2013)	El-Hattab et al. (2016)	Sharkia et al. (2019)	Salehi Chalestori et al. (2018)	Thomas et al. (2019)	Ramos et al. (2020)	Yahia et al. (2020)	Chopra et al.(2022)	AlAbdi et al. (2023)	Current study
**No. of Families/Patients**	8 Fa/24 P	11 Fa/15 P	1 Fa/2 P	1 Fa/1 P	1 Fa/2 P	1 Fa/3 P	1 Fa/2 P	1 Fa/1 P	3 Fa/9 P	16 Fa/24 P
**Ethnicity**	Saudi Arabian	Saudi Arabian Yemeni	Arab (Israeli citizen)	Iranian	European	Swedish	Sudanese	Kuwaiti	Saudi Arabian	Egyptian
**Consanguinity**	+	+	+	+	−	−	+	+	NA	+
***ADAT3*** **variants**	c.430G > A (p.Val144Met)	c.430G > A (p.Val144Met)	c.430G > A (p.Val144Met)	c.99_106dup (p.Glu36Glyfs*44)	c.587 C > T (p.Ala196Val), c.586_587delinsTT (p.Ala196Leu)	c.587 C > T (p.Ala196Val), c.820 C > T (p.Gln274*)	c.430G > A (p.Val144Met)	c.430G > A (p.Val144Met)	c.430G > A (p.Val144Met)	c.430G > A (p.Val144Met), c.319G > A (p.Glu107Lys), c.1013_1018dup (p.Arg338_Ile339dup)
**Zygosity**	Homozygous				Compound Heterozygpous		Homozygous			
**Gender**	12 M, 12 F	8 M, 7 F	1 M, 1 F	1 F	1 M, 1 F	1 M, 2 F	2 F	1 M	6 M, 3 F	8 M, 16 F
**Age**	2-24y	1-24y	14/15y	6y	7,12y	4,9,13y	30,34y	9y	NA	10m-40y
**Microcephaly**	11/24	11/15	2/2	+	1/2	3/3	ND	0/1	5/5	12/24
**Growth retardation**	22/24	11/15	2/2	ND	2/2	3/3	2/2	1/1	9/9	16/24
**Strabismus/Esotropia**	22/24	10/15	2/2	0/1	2/2	3/3	2/2	0/1	7/9	24/24
**Dysmorphic facies**	ND	7/15	2/2	1/1	2/2	3/3	2/2	1/1	9/9	24/24
**Intecllectual disability**	24/24	15/15	2/2	1/1	2/2	3/3	2/2	1/1	8/8	24/24
**Motor Delay**	ND	15/15	2/2	ND	2/2	2/2	2/2	ND	9/9	24/24
**Language delay**	ND	15/15	2/2	ND	2/2	3/3	2/2	2/2	8/8	24/24
**Hypotonia**	10/24	6/15	2/2	ND	2/2	2/2	0/2	0/1	5/5	11/24
**Epilepsy**	3/24	3/15	0/2	ND	2/2	2/2	ND	0/1	3/4	5/24
**Corpus callosum hypogenesis**	ND	3/13	2/2	ND	0/1	0/2	0/2	1/1	3/6	22/24

F: Female, Fa: Family, M: Male, P: Patient, ND: Not determined.

Unusual rare findings noted in our patients were macrodontia, delayed teeth eruption, and pectus carinatum. On the contrary, clubfeet, finger and wrist contractures, neuropathy, bullet-shaped distal phalanges, and feeding difficulties were not spotted in any of our patients, although they were reported in patients with *ADAT3* variants^4,5,11,13^.

Strikingly, brain MRI showed the prevalence of corpus callosum abnormalities, which ranged from complete agenesis of the corpus callosum to splenium hypogenesis and dysgenesis of the body in 22/24 patients (91%). Concerning the reported patients with available brain imaging data, corpus callosum abnormalities were less common and detected in 9/27 patients (Table 2). Defective myelination was found in 66.6%, highlighting the crucial role of ADAT3 activity in white matter development. Of note, one of our patients had mild vermian and brainstem hypoplasia which were not recorded in previous reports.

To date, only five different *ADAT3* variants have been described. They are three missense [c.430G > A (p.Val144Met), c.587 C > T (p.Ala196Val), and c.586_587delinsTT (p.Ala196Leu)], one nonsense (c.820 C > T, p.Gln274*), and one frameshift variant (c.99_106dup, p.Glu36Glyfs*44) (Fig. 3B). The c.430G > A (p.Val144Met), which results from the substitution of valine by methionine at position 144 of the protein, is the most prevalent variant found in almost 90% of patients. This variant was initially described in several Saudi Arabian families and was found to have a carrier frequency of 1%^4,5,27^. Subsequent studies reported the p.Val144Met in sporadic cases from Yemen, Arab Israel, Kuwait, and Sudan^11,13^. Interestingly, the remaining two missense variants (p.Ala196Val and p.Ala196Leu) affect codon 196 suggesting that alterations at this residue disrupt ADAT3 function and contribute to the neurodevelopmental phenotype^12^. Of note, the p.Ala196Val has been described in the heterozygous state along with another variant in two unrelated patients from Europe^12,28^. Other *ADAT3* variants (p.Glu36Glyfs*44, p.Ala196Leu, and p.Gln274*) are very rare and found in one patient each.

In the current study, we described 24 new patients from Egypt harboring homozygous *ADAT3* variants. Interestingly, a homogenous mutational pattern was observed in our cohort, as only three variants were identified. Of them, the c.430G > A (p.Val144Met) was identified in 17/24 patients with a frequency of 70%. The high frequency of the p.Val144Met in our population is in accordance with previous reports^4,5^ and demonstrates that it is very likely to have a founder effect in the Middle East and Arab countries. However, the identification of additional cases from other countries in this region will help in confirming this assumption.

In addition to the p.Val144Met, we identified two new variants, c.1013_1018dup (p.Arg338_Ile339dup) and c.319G > A (p.Glu107Lys). The c.1013_1018dup (p.Arg338_Ile339dup), which is an inframe duplication of two amino acids, was found in a single patient. This variant affects the CMP/dCMP-type deaminase domain which is very crucial for ADAT3 function. To our knowledge, this is the first inframe indel variant to be reported in patients with *ADAT3* variants. Overall, no homozygous inframe indel variants are present in gnomAD v.4 database supporting the pathogenicity of our variant.

The c.319G > A (p.Glu107Lys) variant was found in 6 patients from 4 unrelated families (frequency 25%). The relatively high frequency of this variant in our population led us to suspect the presence of a founder effect which was further confirmed by the presence of a shared homozygous region of 3.89 Mb (from chr19:625,198 to chr19:4,511,647) encompassing the *ADAT3* in patients carrying this variant. The c.319G > A (p.Glu107Lys) is not present in all public genetic databases or our inhouse database and affects a moderately conserved residue. Most in silico prediction tools (which rely mainly on parameters such as evolutionary conservation, sequence homology, and physical properties of the amino acids) showed conflicting interpretations of its pathogenicity^15,20^. However, studying the variant effect on the protein structure and stability postulated negative impacts^23,24^. Furthermore, a predicted slight change in MFE score of the mutant mRNA might affect the RNA stability (http://rna.tbi.univie.ac.at//cgi-bin/RNAWebSuite/RNAfold.cgi). Overall, the c.319G > A (p.Glu107Lys) is suggested to negatively impact the natural structure and function of ADAT3. However, it needs to be further validated through laboratory experiments to confirm its role in the pathophysiology of the disease.

The correlation between *ADAT3* variants and the clinical phenotype remains complex due to the broad phenotypic spectrum and variable expressivity observed among affected individuals as well as the limited number of reported variants. Missense variants often lead to strabismus and moderate to severe intellectual disability^7,28^. A more severe phenotype was noticed in a patient with the nonsense variant p.Gln274* along with p.Ala196Val, likely due to complete loss of protein function which affects ADAT2/ADAT3 binding. However, a patient with a homozygous frameshift variant (p.Glu36Glyfs*44) was associated with a milder phenotype^10^. Therefore, phenotype-genotype correlations appear difficult to establish and the involvement of additional factors such as epigenetic factors or gene modifiers cannot be ruled out.

## Conclusion

We described a large cohort of patients with homozygous *ADAT3* variants and highlighted the clinical and brain imaging characteristics. The constellation of strabismus, dysmorphic facies, intellectual disability, and corpus callosum hypogenesis appears pathognomonic and points to the diagnosis of *ADAT3*-related disorder. The high frequency of the c.430G > A (p.Val144Met) confirms previous reports and suggests the presence of a founder effect in the Middle East and Arab region. We also identified two new variants, including a unique founder Egyptian variant expanding the mutational spectrum of the disorder and enriching population genetic databases.

## Electronic supplementary material

Below is the link to the electronic supplementary material.

Supplementary Material 1

## Data Availability

Data Availability: The data that support the findings of this study are available upon request from the corresponding author.

## Declarations

Helsinki’s guiding principles were followed during the study conduct. The study has been approved by NRC Medical Research Ethics Committee of NRC and written informed consents were obtained from the parents.
