# Supplementary material for: ADAT3-related neurodevelopmental disorder in 24 new patients with a high frequency of the p.Val144Met and a new founder variant
Source: Sci Rep. 2025 Jun 27;15:20329. doi: 10.1038/s41598-025-06857-2 (PMC12205074; doi:10.1038/s41598-025-06857-2)
Supplement: Supplementary file 1 — Supplementary Material 1 [file 41598_2025_6857_MOESM1_ESM.docx]

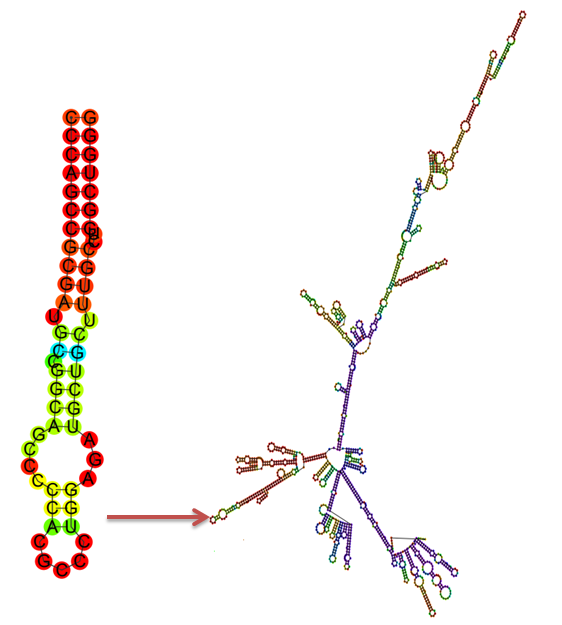

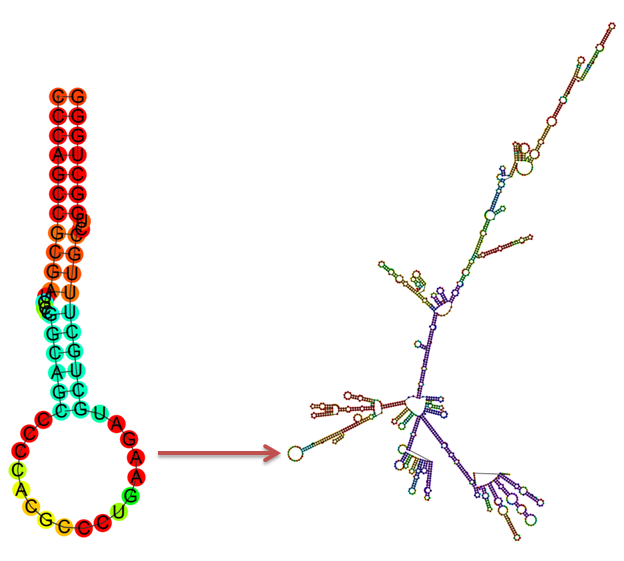


**(B)**

**(A)**

**Wild type c.319G>A**

**c.319G>A**

**Fig. S1:** (A) close up of the ADAT3 mRNA secondary structure ( wild type) (MFE score= -788), (B) close up of the mutant ADAT3 mRNA secondary structure (c.319G>A) (MFE score= -785) ([Gruber et al. 2015](#_ENREF_1)).
